# Supplementary material for: Inverse and Proportional Trans Modulation of Gene Expression in Human Aneuploidies
Source: Genes (Basel). 2024 May 17;15(5):637. doi: 10.3390/genes15050637 (PMC11121296; doi:10.3390/genes15050637)
Supplement: Supplementary file 1 [file genes-15-00637-s001.zip › File S1.pdf]

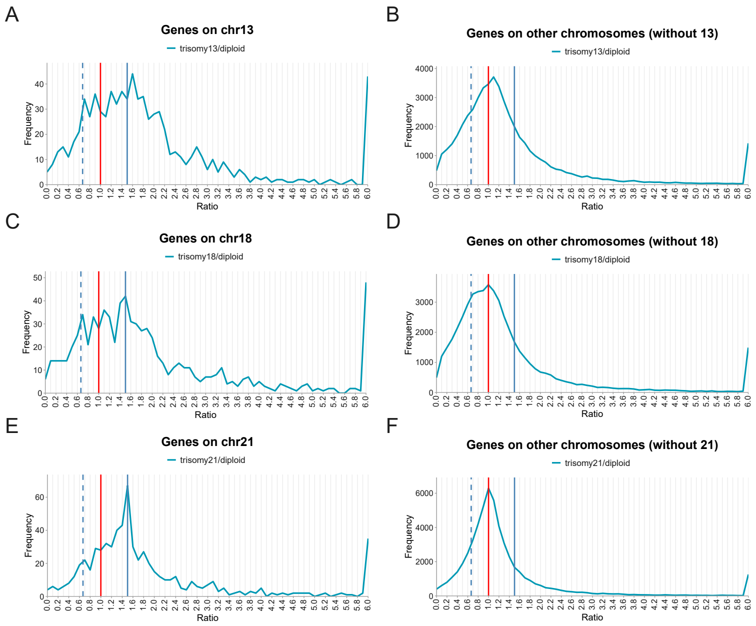

Figure S1. Ratio distributions of gene expression generated using TPM in human autosomal aneuploidies. The ratio distributions of *cis* and *trans* genes in trisomy 13 (**A** and **B**), trisomy 18 (**C** and **D**), and trisomy 21 (**E** and **F**) are shown, respectively. The X-axis indicates the ratio of gene expression, and the Y-axis indicates the frequency of the ratios that fall into each bin of 0.1. The red vertical line represents the ratio of 1.00, and the blue vertical dashed and solid lines indicate 0.67 and 1.5, respectively.

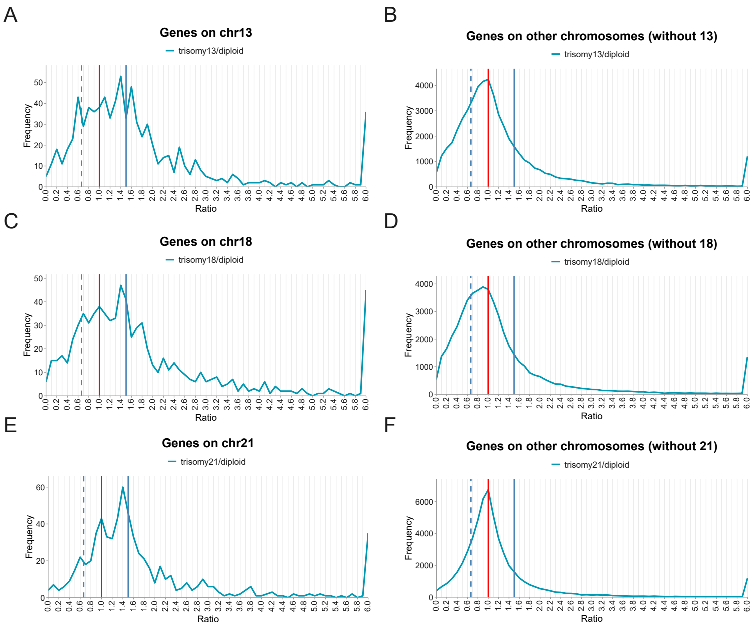

Figure S2. Ratio distributions of gene expression generated using TMM in human autosomal aneuploidies. The ratio distributions of *cis* and *trans* genes in trisomy 13 (**A** and **B**), trisomy 18 (**C** and **D**), and trisomy 21 (**E** and **F**) are shown, respectively. The X-axis indicates the ratio of gene expression, and the Y-axis indicates the frequency of the ratios that fall into each bin of 0.1. The red vertical line represents the ratio of 1.00, and the blue vertical dashed and solid lines indicate 0.67 and 1.5, respectively.

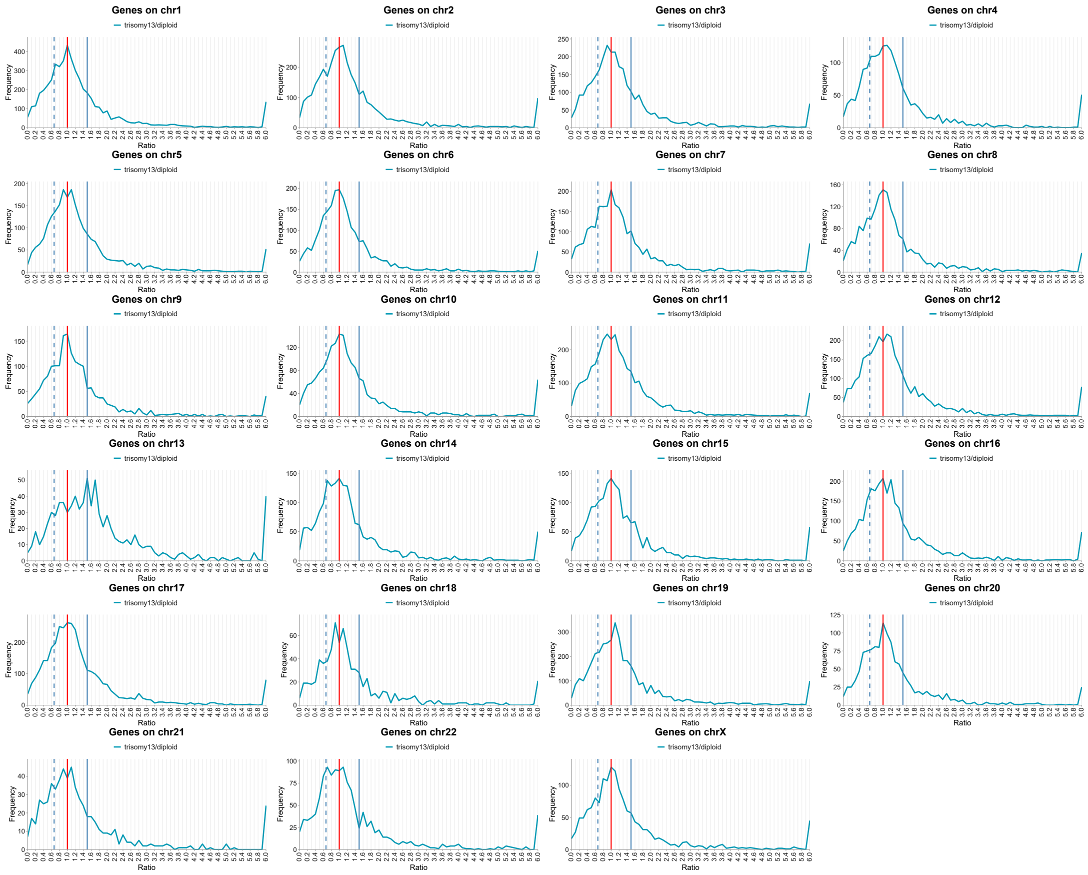

Figure S3. Ratio distributions of gene expression on each chromosome in trisomy 13 individuals. CPM values were used for analysis. The X-axis indicates the ratio of gene expression, and the Y-axis indicates the frequency of the ratios that fall into each bin of 0.1. The red vertical line represents the ratio of 1.00, and the blue vertical dashed and solid lines indicate 0.67 and 1.5, respectively.

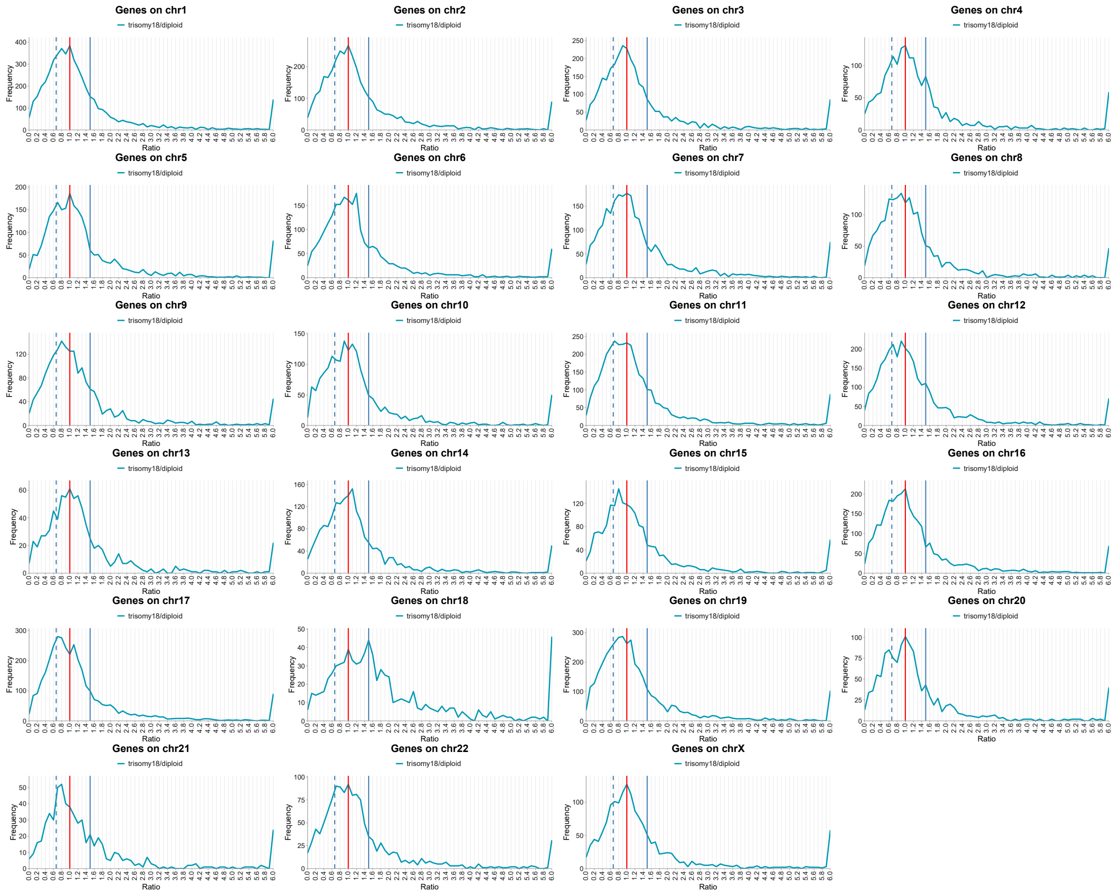

Figure S4. Ratio distributions of gene expression on each chromosome in trisomy 18 individuals. CPM values were used for analysis. The X-axis indicates the ratio of gene expression, and the Y-axis indicates the frequency of the ratios that fall into each bin of 0.1. The red vertical line represents the ratio of 1.00, and the blue vertical dashed and solid lines indicate 0.67 and 1.5, respectively.

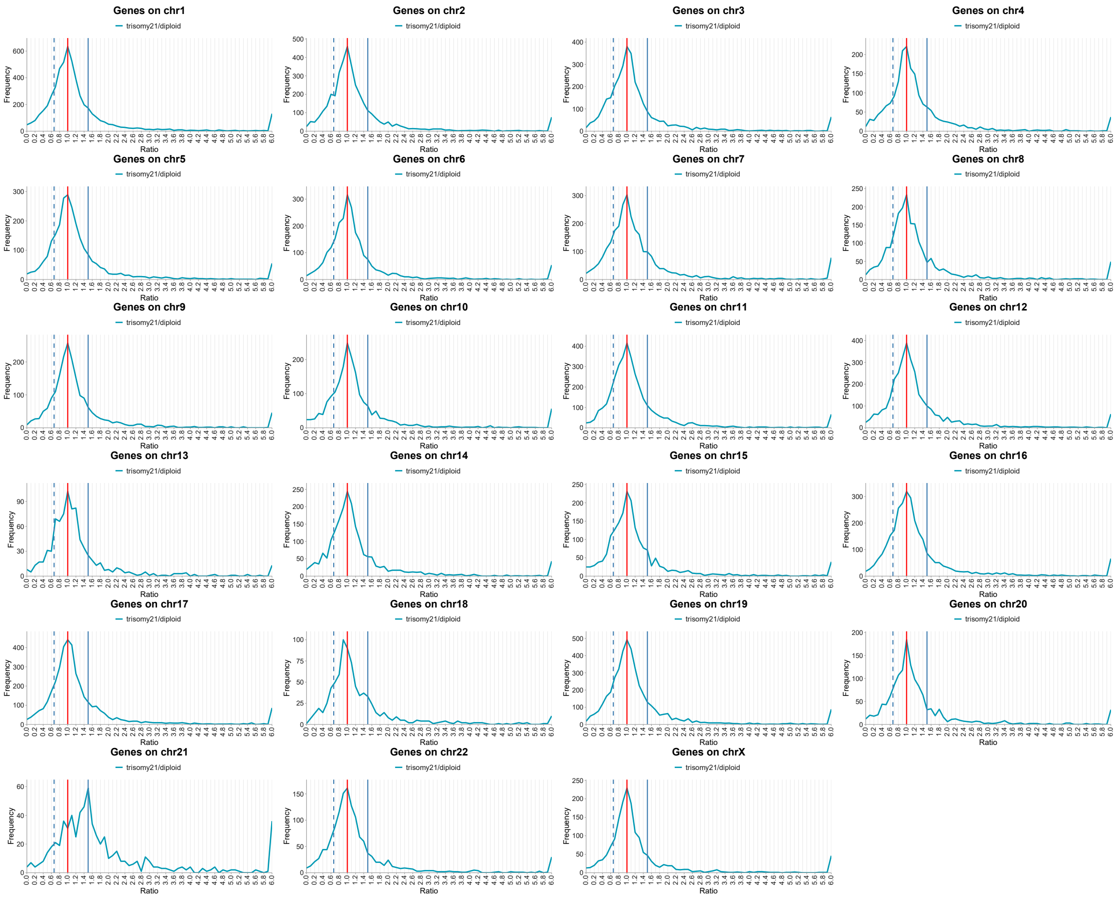

Figure S5. Ratio distributions of gene expression on each chromosome in trisomy 21 individuals. CPM values were used for analysis. The X-axis indicates the ratio of gene expression, and the Y-axis indicates the frequency of the ratios that fall into each bin of 0.1. The red vertical line represents the ratio of 1.00, and the blue vertical dashed and solid lines indicate 0.67 and 1.5, respectively.

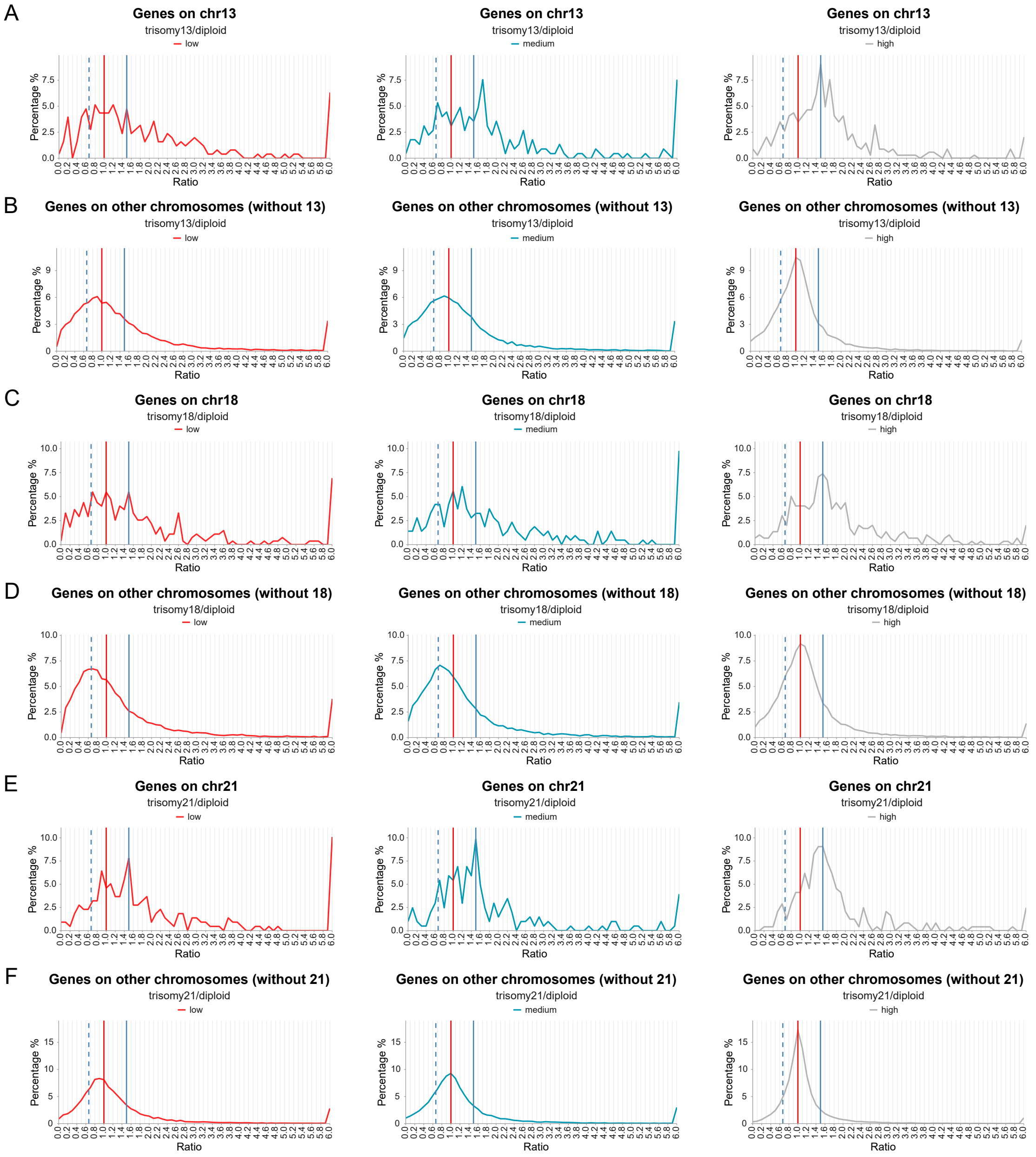

Figure S6. Ratio distribution analysis of genes with different expression levels in human autosomal aneuploidies. The ratio distributions of low, medium, and high expressed *cis* and *trans* genes in trisomy 13 (**A** and **B**), trisomy 18 (**C** and **D**), and trisomy 21 (**E** and **F**) are shown, respectively. The X-axis indicates the ratio of gene expression, and the Y-axis indicates the frequency of the ratios that fall into each bin of 0.1. The red vertical line represents the ratio of 1.00, and the blue vertical dashed and solid lines indicate 0.67 and 1.5, respectively. For low, medium and high expression genes, mean counts range from 5-20, 20-100, and >100, with each group containing approximately one-third of the total transcripts.

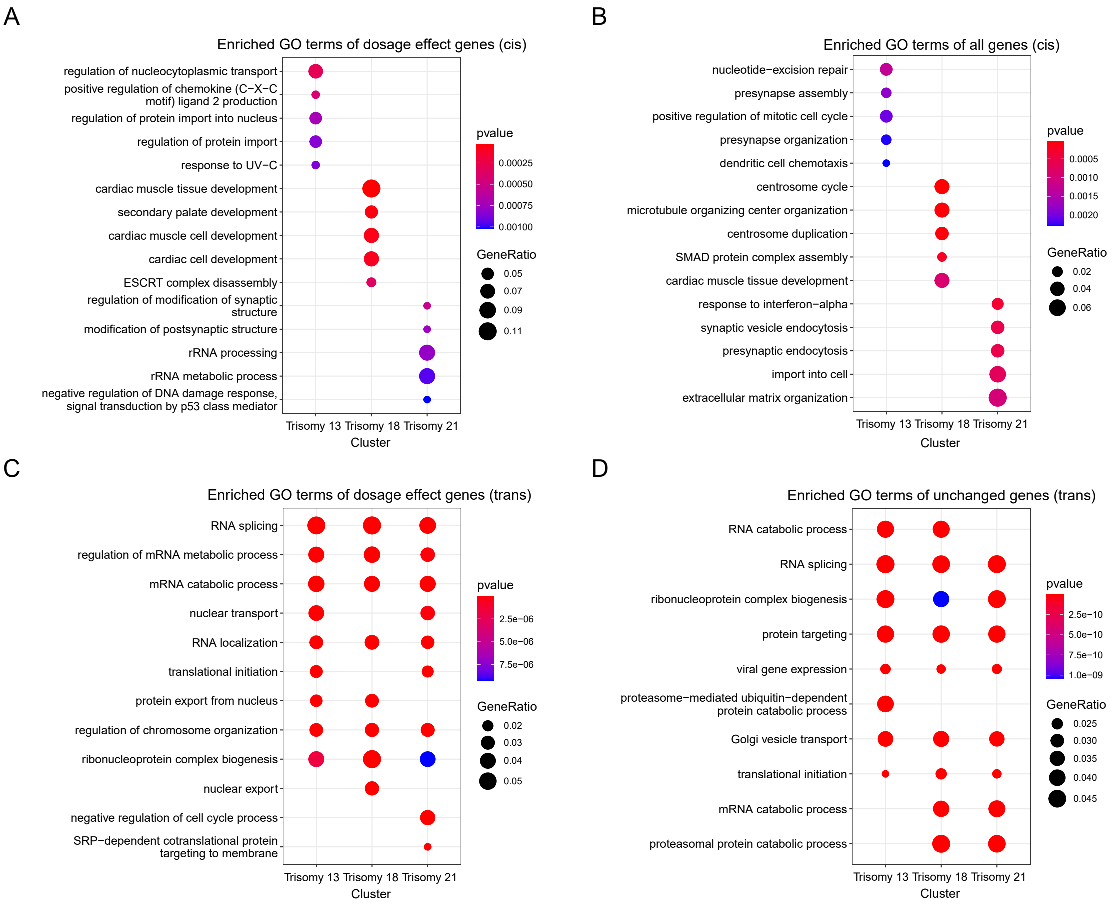

Figure S7. Enriched GO terms of different sets of genes in three kinds of autosomal aneuploidies. (**A**) Functional enrichment analysis of *cis* genes showing a dosage effect (ratios between 1.4 and 1.6). (**B**) Functional enrichment analysis of all *cis* genes in each aneuploidy. (**C**) Functional enrichment analysis of *trans* genes showing proportional dosage effect (ratios between 1.4 and 1.6). (**D**) Functional enrichment analysis of unchanged *trans* genes (ratios between 0.9 and 1.1). The size of the bubble represents the gene ratio, and the color represents the p-value as shown in the legend. GO functions in biological process are analyzed. The top five enriched terms are shown, and the same enriched functions between different comparisons are also shown.

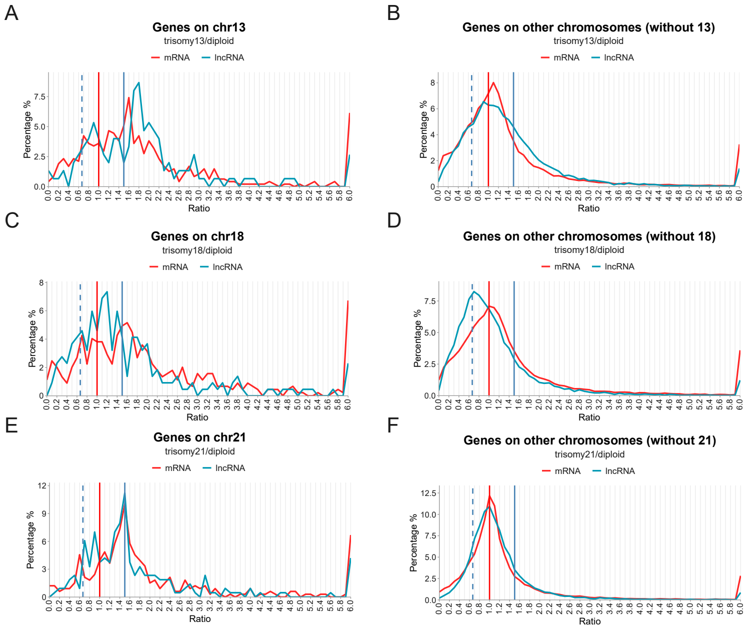

Figure S8. Ratio distributions of lncRNA and mRNA generated using TPM in human autosomal aneuploidies. The ratio distributions of *cis* and *trans* lncRNA (blue lines) and mRNA (red lines) in trisomy 13 (**A** and **B**), trisomy 18 (**C** and **D**), and trisomy 21 (**E** and **F**) are shown, respectively. The X-axis indicates the ratio of gene expression, and the Y-axis indicates the frequency of the ratios that fall into each bin of 0.1. The red vertical line represents the ratio of 1.00, and the blue vertical dashed and solid lines indicate 0.67 and 1.5, respectively.

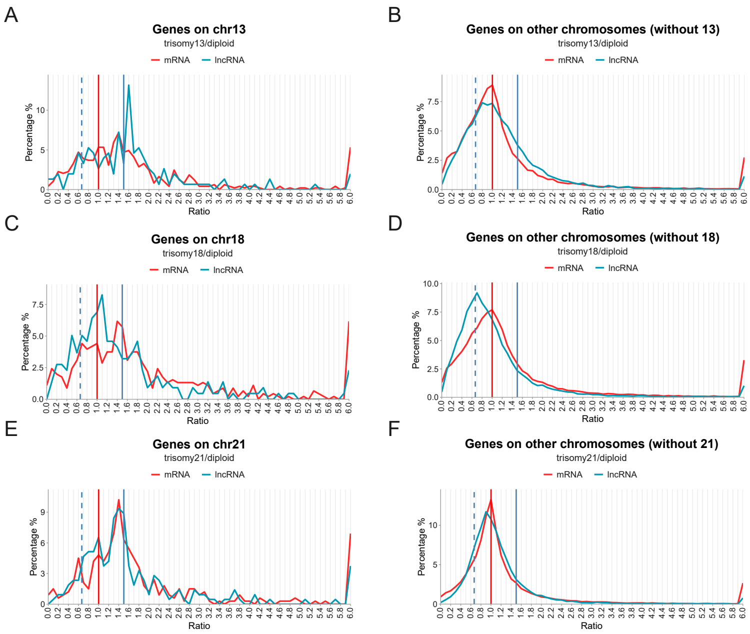

Figure S9. Ratio distributions of lncRNA and mRNA generated using TMM in human autosomal aneuploidies. The ratio distributions of *cis* and *trans* lncRNA (blue lines) and mRNA (red lines) in trisomy 13 (**A** and **B**), trisomy 18 (**C** and **D**), and trisomy 21 (**E** and **F**) are shown, respectively. The X-axis indicates the ratio of gene expression, and the Y-axis indicates the frequency of the ratios that fall into each bin of 0.1. The red vertical line represents the ratio of 1.00, and the blue vertical dashed and solid lines indicate 0.67 and 1.5, respectively.

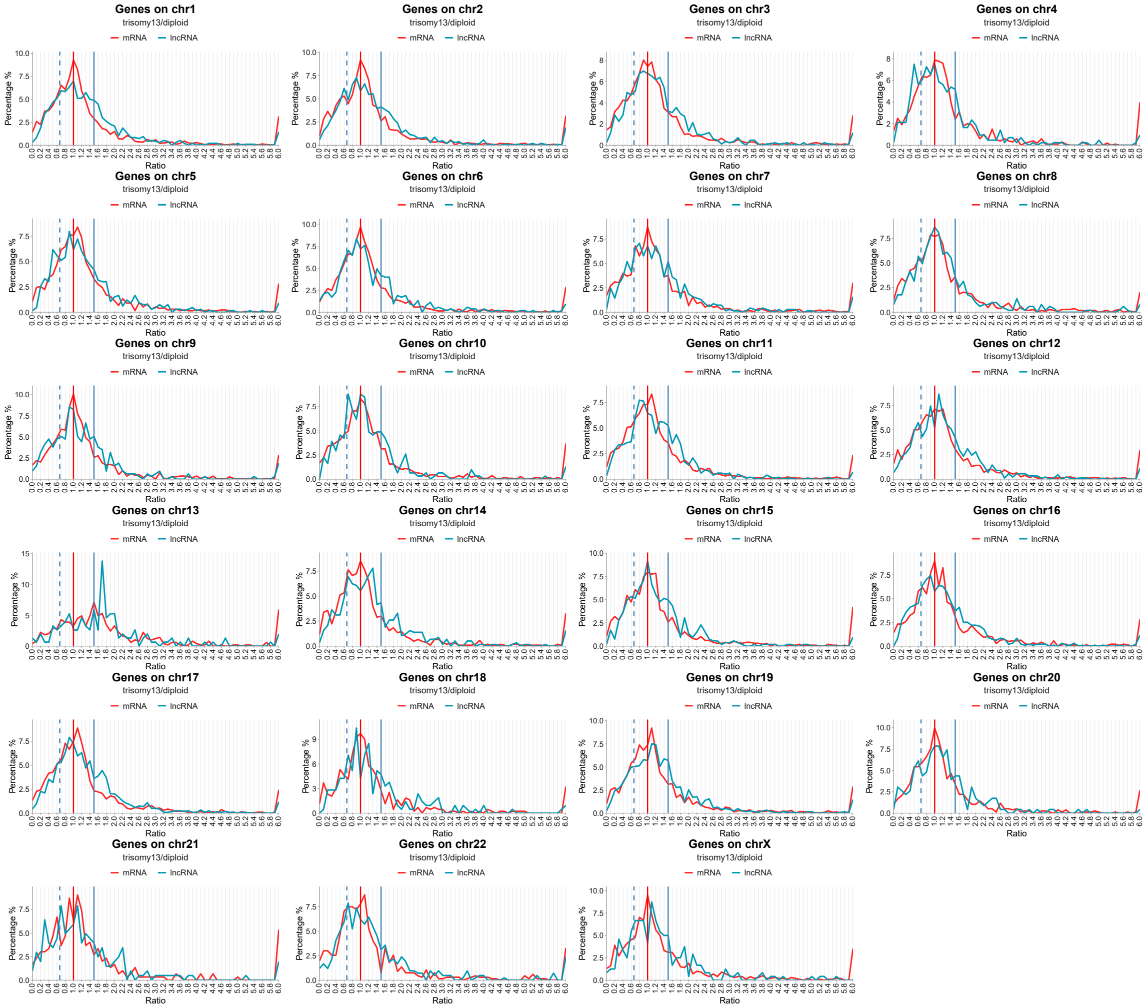

Figure S10. Ratio distributions of lncRNA and mRNA on each chromosome in trisomy 13 individuals. CPM values were used for ratio distribution analysis. The X-axis indicates the ratio of gene expression, and the Y-axis indicates the frequency of the ratios that fall into each bin of 0.1. The red vertical line represents the ratio of 1.00, and the blue vertical dashed and solid lines indicate 0.67 and 1.5, respectively.

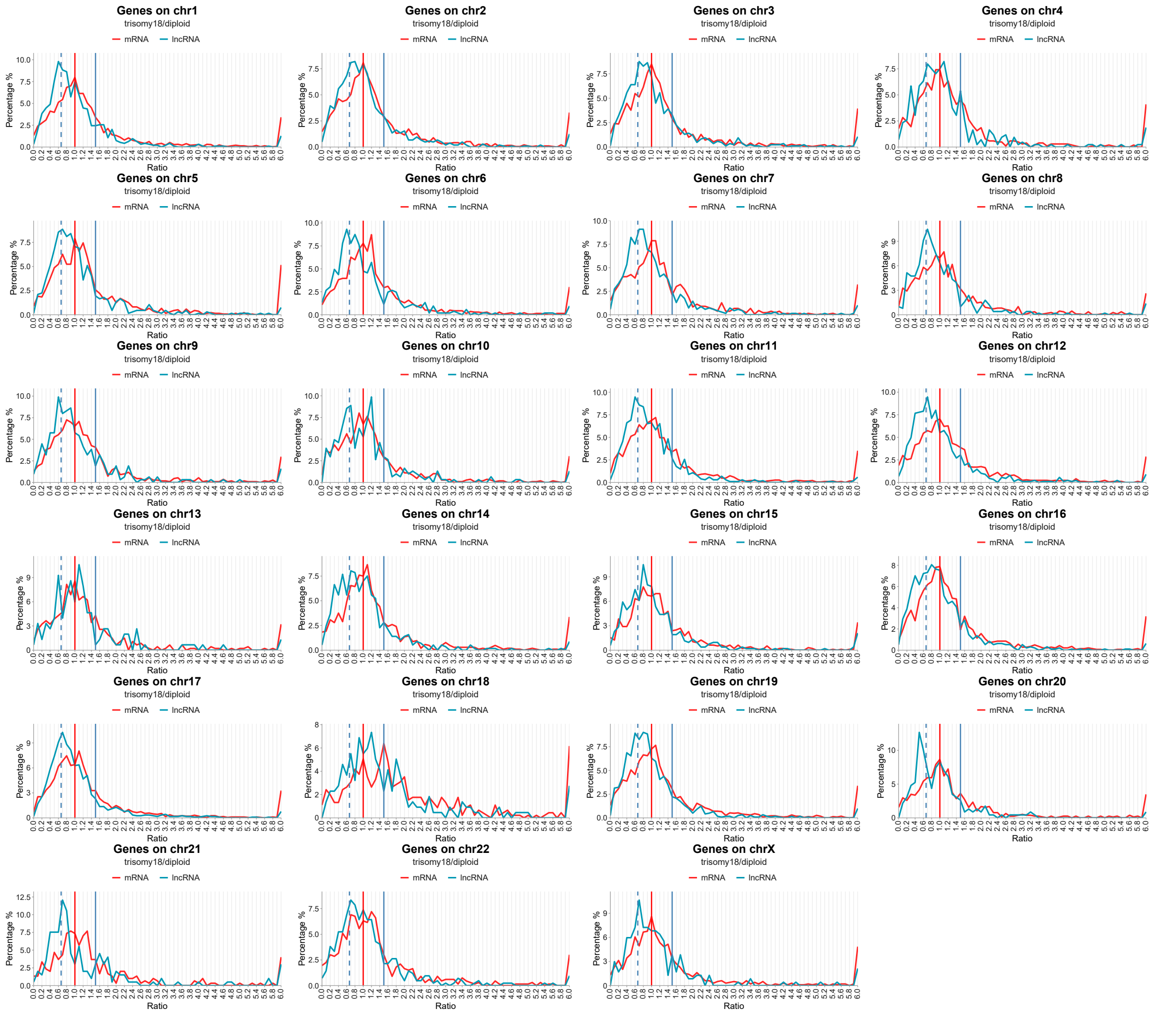

Figure S11. Ratio distributions of lncRNA and mRNA on each chromosome in trisomy 18 individuals. CPM values were used for ratio distribution analysis. The X-axis indicates the ratio of gene expression, and the Y-axis indicates the frequency of the ratios that fall into each bin of 0.1. The red vertical line represents the ratio of 1.00, and the blue vertical dashed and solid lines indicate 0.67 and 1.5, respectively.

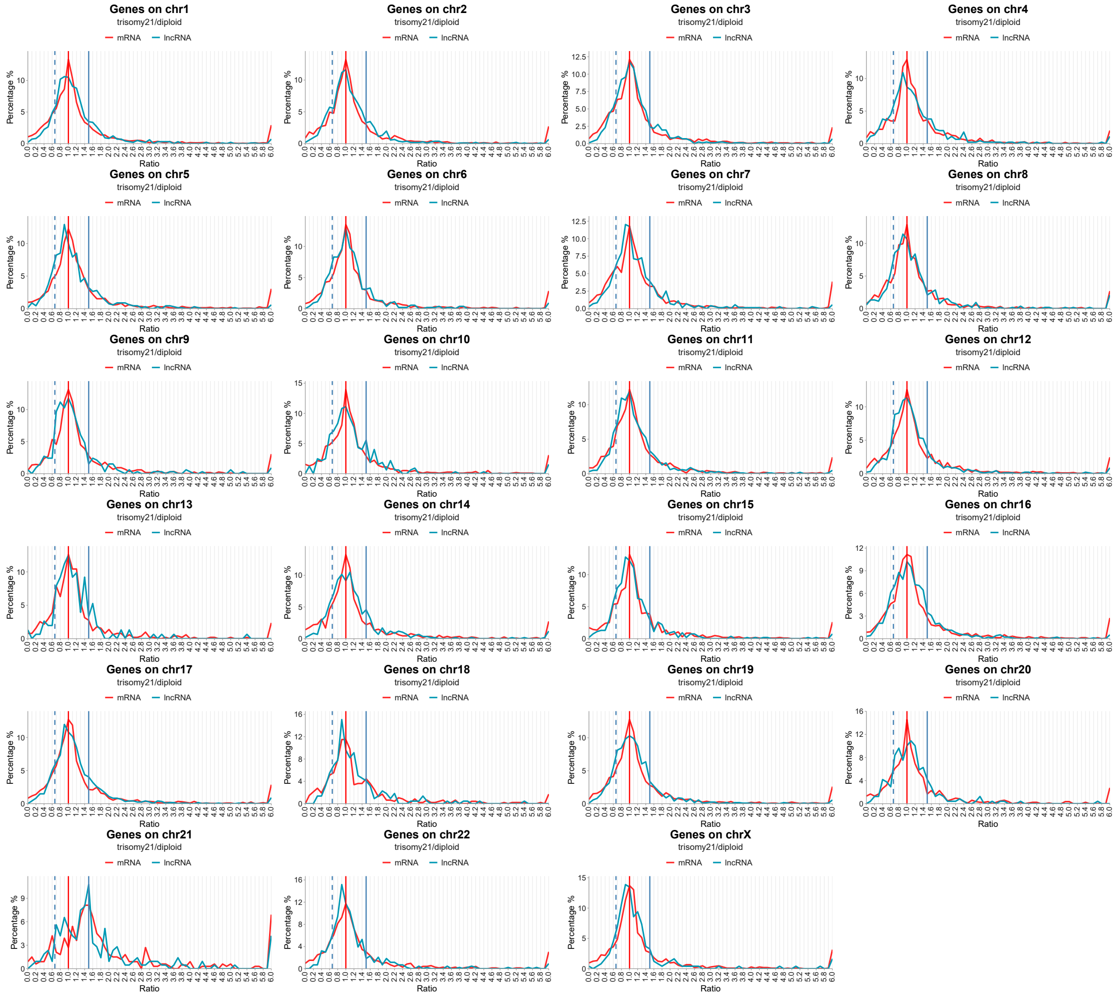

Figure S12. Ratio distributions of lncRNA and mRNA on each chromosome in trisomy 21 individuals. CPM values were used for ratio distribution analysis. The X-axis indicates the ratio of gene expression, and the Y-axis indicates the frequency of the ratios that fall into each bin of 0.1. The red vertical line represents the ratio of 1.00, and the blue vertical dashed and solid lines indicate 0.67 and 1.5, respectively.

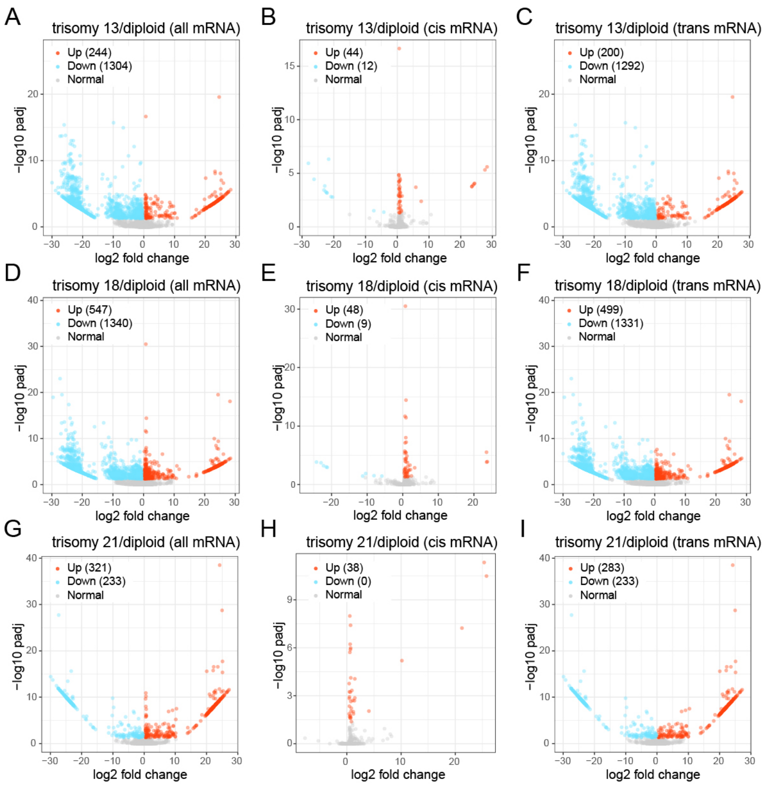

Figure S13. Volcano plots of differentially expressed mRNA (DE-mRNA) in human autosomal aneuploidies. (**A**-**C**) Volcano plots of DE-mRNA in trisomy 13 compared with normal diploid. (**D**-**F**) Volcano plots of DE-mRNA in trisomy 18 compared with normal diploid. (**G**-**I**) Volcano plots of DE-mRNA in trisomy 21 compared with normal diploid. Genes located on all chromosomes (**A**, **D** and **G**), *cis* mRNA (**B**, **E** and **H**), and *trans* mRNA (**C**, **F** and **I**) are shown respectively. DE-mRNAs are defined as adjusted p-value < 0.05. Significantly up-regulated mRNAs are represented by red dots and significantly down-regulated mRNAs are represented by blue dots. The legend in the upper left corner shows the number of up-regulated and down-regulated transcripts in each plot.

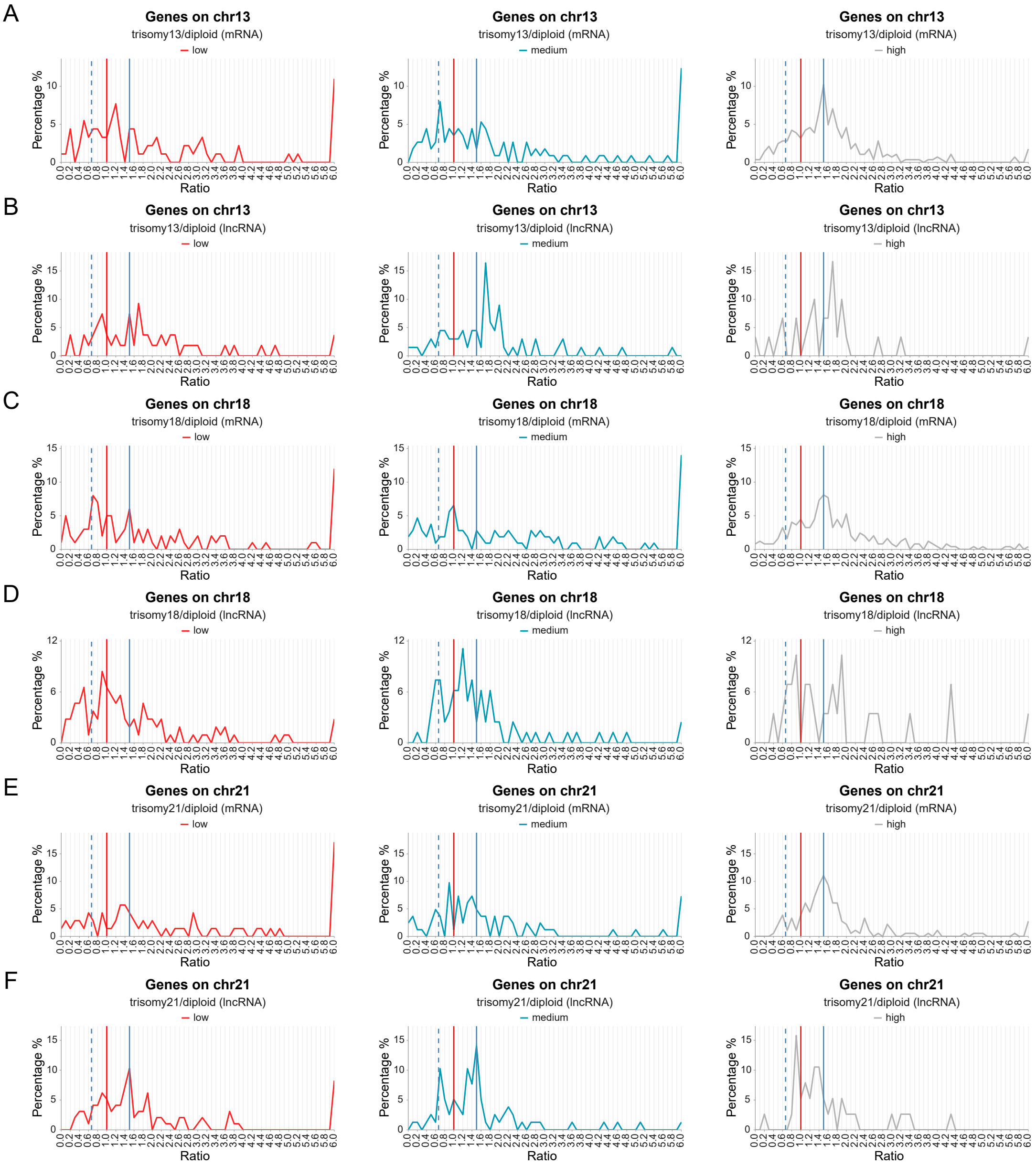

Figure S14. Ratio distribution analysis of *cis* mRNA and lncRNA with different expression levels in aneuploidies. The ratio distributions of low, medium, and high expressed *cis* mRNA and lncRNA in trisomy 13 (**A** and **B**), trisomy 18 (**C** and **D**), and trisomy 21 (**E** and **F**) are shown, respectively. The X-axis indicates the ratio of gene expression, and the Y-axis indicates the frequency of the ratios that fall into each bin of 0.1. The red vertical line represents the ratio of 1.00, and the blue vertical dashed and solid lines indicate 0.67 and 1.5, respectively. For low, medium, and high expression genes, mean counts ranges from 5-20, 20-100, and >100.

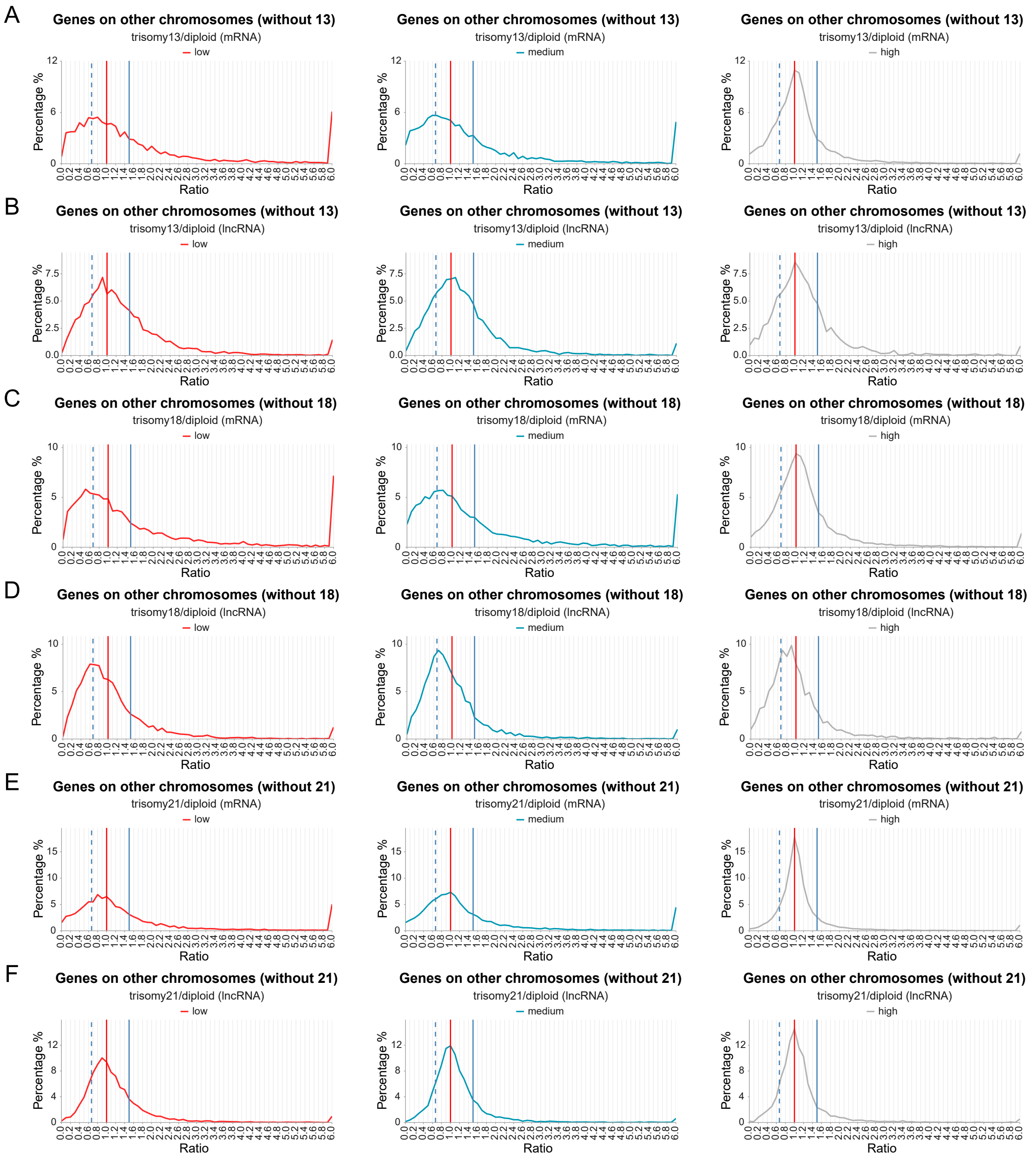

Figure S15. Ratio distribution analysis of *trans* mRNA and lncRNA with different expression levels in aneuploidies. The ratio distributions of low, medium, and high expressed *trans* mRNA and lncRNA in trisomy 13 (**A** and **B**), trisomy 18 (**C** and **D**), and trisomy 21 (**E** and **F**) are shown, respectively. The X-axis indicates the ratio of gene expression, and the Y-axis indicates the frequency of the ratios that fall into each bin of 0.1. The red vertical line represents the ratio of 1.00, and the blue vertical dashed and solid lines indicate 0.67 and 1.5, respectively. For low, medium, and high expression genes, mean counts ranges from 5-20, 20-100, and >100.

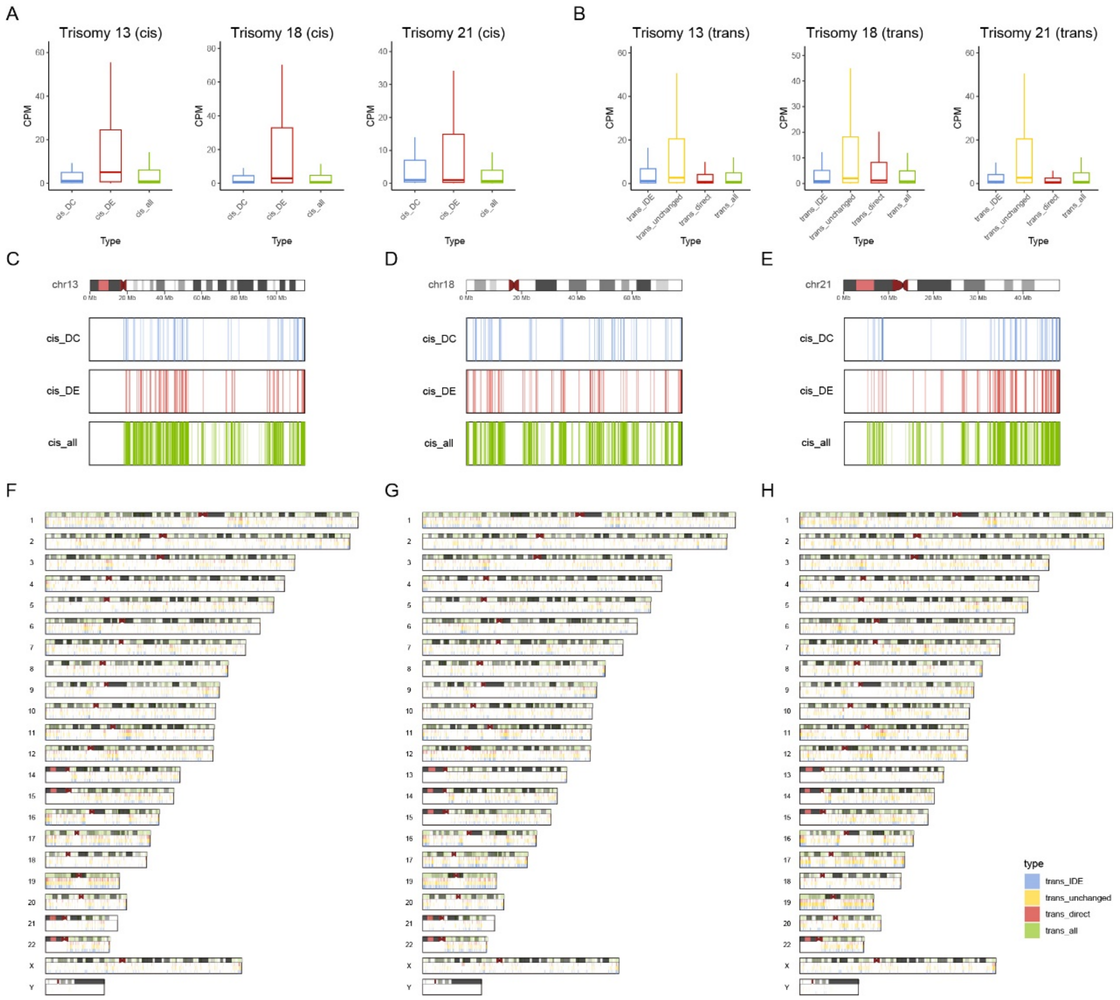

Figure S16. There was no strong correlation between gene expression changes and basal expression levels or gene locations. (**A**-**B**) Boxplots of basal expression levels of *cis* (**A**) and *trans* (**B**) genes with different expression patterns in normal diploids. (**C**-**E**) Distributions of *cis* genes with different expression patterns on varied chromosomes in trisomy 13 (**C**), 18 (**D**), and 21 (**E**). (**F**-**H**) Distributions of *trans* genes with different expression patterns on varied chromosomes in trisomy 13 (**F**), 18 (**G**), and 21 (**H**).
