# Supplementary material for: Inverse and Proportional Trans Modulation of Gene Expression in Human Aneuploidies
Source: Genes (Basel). 2024 May 17;15(5):637. doi: 10.3390/genes15050637 (PMC11121296; doi:10.3390/genes15050637)
Supplement: Supplementary file 1 [file genes-15-00637-s001.zip › File S2.pdf]

Table S1. The number of all differentially expressed genes.

		all-transcripts			cis-transcripts			trans-transcripts		
		all	mRNA	lncRNA	all	mRNA	lncRNA	all	mRNA	lncRNA
trisomy13	up	380	244	69	51	44	4	329	200	65
	down	1677	1304	120	14	12	1	1663	1292	119
	normal	45694	28191	10310	675	426	120	45019	27765	10190
trisomy18	up	689	547	53	58	48	5	631	499	48
	down	1928	1340	285	10	9	0	1918	1331	285
	normal	45134	27852	10161	588	369	150	44546	27483	10011
trisomy21	up	446	321	53	53	38	13	393	283	40
	down	320	233	31	0	0	0	320	233	31
	normal	46985	29185	10415	515	272	153	46470	28913	10262

The number of all differentially expressed genes (DEGs, adjusted p-value < 0.05) in human autosomal aneuploidy trisomy 13, trisomy 18, and trisomy 21 is shown in the table.

Table S2. The number of low expressed DEGs.

		all-transcripts			cis-transcripts			trans-transcripts		
		all	mRNA	lncRNA	all	mRNA	lncRNA	all	mRNA	lncRNA
trisomy13	up	38	14	16	1	1	0	37	13	16
	down	192	123	27	0	0	0	192	123	27
	normal	9341	3717	3311	137	57	27	9204	3660	3284
trisomy18	up	43	22	11	2	1	1	41	21	10
	down	243	123	65	2	2	0	241	121	65
	normal	9285	3709	3278	118	51	42	9167	3658	3236
trisomy21	up	63	38	13	2	0	2	61	38	11
	down	60	43	4	0	0	0	60	43	4
	normal	9448	3773	3337	109	39	47	9339	3734	3290

The number of DEGs with low expression (mean counts > 5 and < 20, adjusted p-value < 0.05) in human autosomal aneuploidy trisomy 13, trisomy 18, and trisomy 21 is shown in the table.

Table S3. The number of medium expressed DEGs.

		all-transcripts			cis-transcripts			trans-transcripts		
		**all**	**mRNA**	**lncRNA**	**all**	**mRNA**	**lncRNA**	**all**	**mRNA**	**lncRNA**
trisomy13	up	200	129	37	9	7	2	191	122	35
	down	725	534	55	9	8	1	716	526	54
	normal	15816	7822	4818	233	119	65	15583	7703	4753
trisomy18	up	232	161	25	9	5	3	223	156	22
	down	812	512	132	5	4	0	807	508	132
	normal	15697	7812	4753	216	111	79	15481	7701	4674
trisomy21	up	221	153	24	12	4	7	209	149	17
	down	146	101	15	0	0	0	146	101	15
	normal	16374	8231	4871	199	83	72	16175	8148	4799

The number of DEGs with medium expression (mean counts > 20 and < 100, adjusted p-value < 0.05) in human autosomal aneuploidy trisomy 13, trisomy 18, and trisomy 21 is shown in the table.

Table S4. The number of high expressed DEGs.

		all-transcripts			cis-transcripts			trans-transcripts		
		**all**	**mRNA**	**lncRNA**	**all**	**mRNA**	**lncRNA**	**all**	**mRNA**	**lncRNA**
trisomy13	up	142	101	16	41	36	2	101	65	14
	down	760	647	38	5	4	0	755	643	38
	normal	20537	16652	2181	305	250	28	20232	16402	2153
trisomy18	up	414	364	17	47	42	1	367	322	16
	down	873	705	88	3	3	0	870	702	88
	normal	20152	16331	2130	254	207	29	19898	16124	2101
trisomy21	up	162	130	16	39	34	4	123	96	12
	down	114	89	12	0	0	0	114	89	12
	normal	21163	17181	2207	207	150	34	20956	17031	2173

The number of DEGs with high expression (mean counts > 100, adjusted p-value < 0.05) in human autosomal aneuploidy trisomy 13, trisomy 18, and trisomy 21 is shown in the table.

Table S5. The number of DEGs in human sex chromosome aneuploidy XO and XXY.

		all-transcripts			X-linked transcripts			autosomal transcripts		
		**all**	**mRNA**	**lncRNA**	**all**	**mRNA**	**lncRNA**	**all**	**mRNA**	**lncRNA**
XO/XX	up	280	201	46	12	11	1	268	190	45
	down	330	220	43	32	16	9	298	204	34
	normal	43347	25078	10927	1224	823	173	42088	24239	10747
XXY/XY	up	119	79	14	24	10	8	94	69	6
	down	102	78	8	3	2	0	99	76	8
	normal	43736	25342	10994	1241	838	175	42461	24488	10812
XXY/XX	up	129	87	19	3	3	0	94	69	12
	down	133	92	12	3	1	0	130	91	12
	normal	43695	25320	10985	1262	846	183	42430	24473	10802
XY/XX	up	130	90	17	0	0	0	99	76	10
	down	179	120	28	19	7	8	160	113	20
	normal	43648	25289	10971	1249	843	175	42395	24444	10796

The number of significantly up-regulated and down-regulated transcripts (adjusted p-value < 0.05) in human sex chromosome aneuploidy XO and XXY is shown in the table.

Table S6. The proportion of up- and down-regulated transcripts in sex chromosome aneuploidy XXY, XXXY, and XXXXY.

		down-regulated (ratio < 0.8)				up-regulated (ratio > 1.25)			
		XX/XY	XXY/XY	XXXY/XY	XXXXY/XY	XX/XY	XXY/XY	XXXY/XY	XXXXY/XY
all type	chrX	23.60%	13.76%	37.27%	29.21%	28.46%	24.44%	22.38%	29.78%
	autosomes	24.85%	13.96%	33.68%	28.67%	24.41%	22.13%	25.64%	25.93%
mRNA	chrX	15.21%	9.87%	26.70%	25.89%	22.98%	11.97%	19.09%	21.84%
	autosomes	15.69%	8.54%	23.18%	21.51%	18.40%	10.39%	21.78%	18.72%
lncRNA	chrX	24.91%	13.75%	38.29%	31.97%	21.19%	20.45%	21.93%	23.05%
	autosomes	22.36%	11.58%	29.90%	27.08%	22.55%	20.04%	26.11%	23.63%

The proportion of up-regulated (ratio > 1.25) and down-regulated (ratio < 0.8) transcripts on X chromosome and autosomes in human sex chromosome aneuploidy XXY, XXXY, and XXXXY is shown in the table.

Table S7. The number of up- and down-regulated transcripts in sex chromosome aneuploidy XXY, XXXY, and XXXXY.

		**all-transcripts**			**X-linked transcripts**			**autosomal transcripts**		
		**all**	**mRNA**	**lncRNA**	**all**	**mRNA**	**lncRNA**	**all**	**mRNA**	**lncRNA**
XX/XY	up	3160	2475	1697	136	109	38	2999	2364	1659
	down	2974	1915	1580	122	93	32	2778	1807	1539
	normal	23114	11699	11720	822	416	201	21907	11261	11519
XXY/XY	up	4725	2818	2589	184	110	44	4476	2705	2543
	down	2118	1785	1142	117	112	33	1987	1672	1109
	normal	22694	11488	11311	788	396	195	21487	11057	11109
XXXY/XY	up	2062	1326	1148	59	45	14	1980	1280	1134
	down	4122	2110	2043	167	106	46	3885	2000	1996
	normal	22981	12652	11806	848	467	211	21748	12151	11587
XXXXY/XY	up	3534	2816	1958	143	107	36	3371	2707	1922
	down	4401	3146	2481	198	171	63	4143	2973	2418
	normal	21319	10127	10567	738	340	172	20179	9752	10386

The number of significantly up-regulated and down-regulated transcripts (t-test p-value < 0.05) in human sex chromosome aneuploidy XXY, XXXY, and XXXXY is shown in the table.

Table S8. The number of up- and down-regulated target genes of all TFs in human autosomal aneuploidies.

		**cis-TF (all)**		**trans-TF (all)**	
		**cis-target**	**trans-target**	**cis-target**	**trans-target**
trisomy 13	up	44	344	61	426
	down	12	1432	17	1964
	normal	346	30721	583	40278
trisomy 18	up	2	36	70	830
	down	0	119	11	2277
	normal	28	2181	529	40328
trisomy 21	up	53	394	53	440
	down	0	332	0	375
	normal	377	38837	419	42720

The number of up- and down-regulated (padj < 0.1) target genes of all transcription factors (TFs) in human autosomal aneuploidies trisomy 13, trisomy 18, and trisomy 21 is shown in the table.

Table S9. The number of up- and down-regulated targets of positively changed TFs in human autosomal aneuploidies.

		**cis-TF (> 1.25)**		**trans-TF (> 1.25)**	
		**cis-target**	**trans-target**	**cis-target**	**trans-target**
trisomy 13	up	42	333	60	425
	down	12	1373	17	1938
	normal	334	29765	576	39938
trisomy 18	up	2	33	70	826
	down	0	110	11	2264
	normal	28	2078	526	40165
trisomy 21	up	53	394	51	434
	down	0	332	0	366
	normal	377	38837	406	42247

The number of up- and down-regulated (padj < 0.1) target genes of positively changed TFs (> 1.25) in human autosomal aneuploidy trisomy 13, trisomy 18, and trisomy 21 is shown in the table.

Table S10. The number of up- and down-regulated targets of negatively changed TFs in human autosomal aneuploidies.

		**cis-TF (< 0.8)**		**trans-TF (< 0.8)**	
		**cis-target**	**trans-target**	**cis-target**	**trans-target**
trisomy 13	up	10	165	61	424
	down	4	620	17	1948
	normal	119	13981	580	40110
trisomy 18	up	2	31	70	827
	down	0	105	11	2264
	normal	28	1903	529	40163
trisomy 21	up	12	103	52	434
	down	0	91	0	368
	normal	99	9836	417	42446

The number of up- and down-regulated (padj < 0.1) target genes of negatively changed TFs (< 0.8) in human autosomal aneuploidy trisomy 13, trisomy 18, and trisomy 21 is shown in the table.

Table S11. The number of up- and down-regulated target genes of unchanged TFs in human autosomal aneuploidies.

		**cis-TF (>0.8 & <1.25)**		**trans-TF (>0.8 & <1.25)**	
		**cis-target**	**trans-target**	**cis-target**	**trans-target**
trisomy 13	up	1	42	61	425
	down	1	148	17	1948
	normal	30	3371	583	40037
trisomy 18	up	2	27	70	820
	down	0	94	11	2254
	normal	28	1659	525	40028
trisomy 21	up	42	355	52	437
	down	0	296	0	374
	normal	329	35032	417	42572

The number of up- and down-regulated (padj < 0.1) target genes of unchanged TFs (> 0.8 and < 1.25) in human autosomal aneuploidy trisomy 13, trisomy 18, and trisomy 21 is shown in the table.

Table S12. The results of statistical tests.

**figures**	**distribution or comparison**	**test**	**p-value**
Fig. 1A	trisomy13 cis	normality test	0.0000E+00
Fig. 1B	trisomy13 trans	normality test	0.0000E+00
Fig. 1C	trisomy18 cis	normality test	0.0000E+00
Fig. 1D	trisomy18 trans	normality test	0.0000E+00
Fig. 1E	trisomy21 cis	normality test	0.0000E+00
Fig. 1F	trisomy21 trans	normality test	0.0000E+00
Fig. 1-fig. S6A	trisomy13 cis - low vs high	K-S test	1.9635E-02
Fig. 1-fig. S6B	trisomy13 trans - low vs high	K-S test	0.0000E+00
Fig. 1-fig. S6C	trisomy18 cis - low vs high	K-S test	1.1859E-04
Fig. 1-fig. S6D	trisomy18 trans - low vs high	K-S test	0.0000E+00
Fig. 1-fig. S6E	trisomy21 cis - low vs high	K-S test	3.1638E-02
Fig. 1-fig. S6F	trisomy21 trans - low vs high	K-S test	0.0000E+00
Fig. 1-fig. S6A	trisomy13 cis - medium vs high	K-S test	7.7676E-02
Fig. 1-fig. S6B	trisomy13 trans - medium vs high	K-S test	0.0000E+00
Fig. 1-fig. S6C	trisomy18 cis - medium vs high	K-S test	4.7205E-02
Fig. 1-fig. S6D	trisomy18 trans - medium vs high	K-S test	0.0000E+00
Fig. 1-fig. S6E	trisomy21 cis - medium vs high	K-S test	8.9044E-02

Fig. 1-fig. S6F	trisomy21 trans - medium vs high	K-S test	0.0000E+00
Fig. 1-fig. S6A	trisomy13 cis - low vs medium	K-S test	1.9635E-02
Fig. 1-fig. S6B	trisomy13 trans - low vs medium	K-S test	0.0000E+00
Fig. 1-fig. S6C	trisomy18 cis - low vs medium	K-S test	1.1859E-04
Fig. 1-fig. S6D	trisomy18 trans - low vs medium	K-S test	0.0000E+00
Fig. 1-fig. S6E	trisomy21 cis - low vs medium	K-S test	3.1638E-02
Fig. 1-fig. S6F	trisomy21 trans - low vs medium	K-S test	0.0000E+00
Fig. 2A	trisomy18/diploid - DC genes identified in trisomy13 vs all chr13 genes	K-S test	4.9869E-01
Fig. 2B	trisomy21/diploid - DC genes identified in trisomy13 vs all chr13 genes	K-S test	4.6560E-01
Fig. 2C	trisomy13/diploid - DC genes identified in trisomy18 vs all chr18 genes	K-S test	5.5409E-02
Fig. 2D	trisomy21/diploid - DC genes identified in trisomy18 vs all chr18 genes	K-S test	1.2115E-02
Fig. 2E	trisomy13/diploid - DC genes identified in trisomy21 vs all chr21 genes	K-S test	2.3720E-03
Fig. 2F	trisomy18/diploid - DC genes identified in trisomy21 vs all chr21 genes	K-S test	4.1489E-01
Fig. 2J	trisomy13/diploid - IDE genes identified in trisomy 18 and 21 vs all chr13 genes	K-S test	2.9480E-03
Fig. 2K	trisomy18/diploid - IDE genes identified in trisomy 13 and 21 vs all chr18 genes	K-S test	2.3487E-06
Fig. 2L	trisomy21/diploid - IDE genes identified in trisomy 13 and 18 vs all chr21 genes	K-S test	1.8613E-03
Fig. 2A	trisomy18/diploid - DC genes identified in trisomy13	normality test	1.3650E-05
Fig. 2B	trisomy21/diploid - DC genes identified in trisomy13	normality test	1.1770E-01
Fig. 2C	trisomy13/diploid - DC genes identified in trisomy18	normality test	5.3040E-07
Fig. 2D	trisomy21/diploid - DC genes identified in trisomy18	normality test	1.1370E-01
Fig. 2E	trisomy13/diploid - DC genes identified in trisomy21	normality test	2.8470E-01
Fig. 2F	trisomy18/diploid - DC genes identified in trisomy21	normality test	6.4590E-01
Fig. 2J	trisomy13/diploid - IDE genes identified in trisomy 18 and 21	normality test	7.0380E-09
Fig. 2K	trisomy18/diploid - IDE genes identified in trisomy 13 and 21	normality test	8.4840E-12
Fig. 2L	trisomy21/diploid - IDE genes identified in trisomy 13 and 18	normality test	1.3410E-06
Fig. 3A	trisomy21 twins cis	normality test	6.1850E-08
Fig. 3B	trisomy21 twins trans	normality test	0.0000E+00
Fig. 3C	trisomy21 unrelated individuals cis	normality test	2.9980E-15
Fig. 3D	trisomy21 unrelated individuals trans	normality test	0.0000E+00
Fig. 3E	trisomy21 female 2-3 days old cis	normality test	0.0000E+00
Fig. 3F	trisomy21 female 2-3 days old trans	normality test	0.0000E+00

Fig. 3G	trisomy21 male 1-2 years old cis	normality test	0.0000E+00
Fig. 3H	trisomy21 male 1-2 years old trans	normality test	0.0000E+00
Fig. 3I	trisomy21 female 11-19 years old cis	normality test	2.6610E-11
Fig. 3J	trisomy21 female 11-19 years old trans	normality test	0.0000E+00
Fig. 3K	trisomy21 male 19-21 years old cis	normality test	9.9250E-14
Fig. 3L	trisomy21 male 19-21 years old trans	normality test	0.0000E+00
Fig. 3A-C	trisomy21 cis - twins vs unrelated individuals	K-S test	2.9615E-08
Fig. 3B-D	trisomy21 trans - twins vs unrelated individuals	K-S test	0.0000E+00
Fig. 3E-G	trisomy21 cis - female 2-3 days vs male 1-2 years	K-S test	5.5823E-02
Fig. 3F-H	trisomy21 trans - female 2-3 days vs male 1-2 years	K-S test	0.0000E+00
Fig. 3E-I	trisomy21 cis - female 2-3 days vs female 11-19 years	K-S test	2.0190E-05
Fig. 3F-J	trisomy21 trans - female 2-3 days vs female 11-19 years	K-S test	0.0000E+00
Fig. 3E-K	trisomy21 cis - female 2-3 days vs male 19-21 years	K-S test	4.1559E-05
Fig. 3F-L	trisomy21 trans - female 2-3 days vs male 19-21 years	K-S test	0.0000E+00
Fig. 3G-I	trisomy21 cis - male 1-2 years vs female 11-19 years	K-S test	2.7751E-03
Fig. 3H-J	trisomy21 trans - male 1-2 years vs female 11-19 years	K-S test	0.0000E+00
Fig. 3G-K	trisomy21 cis - male 1-2 years vs male 19-21 years	K-S test	1.7902E-03
Fig. 3H-L	trisomy21 trans - male 1-2 years vs male 19-21 years	K-S test	0.0000E+00
Fig. 3I-K	trisomy21 cis - female 11-19 years vs male 19-21 years	K-S test	8.7020E-01
Fig. 3J-L	trisomy21 trans - female 11-19 years vs male 19-21 years	K-S test	2.2204E-16
Fig. 4A	trisomy13 cis - mRNA vs lncRNA	K-S test	1.8172E-01
Fig. 4B	trisomy13 trans - mRNA vs lncRNA	K-S test	0.0000E+00
Fig. 4C	trisomy18 cis - mRNA vs lncRNA	K-S test	9.0784E-05
Fig. 4D	trisomy18 trans - mRNA vs lncRNA	K-S test	0.0000E+00
Fig. 4E	trisomy21 cis - mRNA vs lncRNA	K-S test	1.7831E-01
Fig. 4F	trisomy21 trans - mRNA vs lncRNA	K-S test	6.6613E-15
Fig. 4-fig. S7A	trisomy13 cis mRNA - low vs high	K-S test	6.3714E-03
Fig. 4-fig. S7C	trisomy18 cis mRNA - low vs high	K-S test	2.5040E-04
Fig. 4-fig. S7E	trisomy21 cis mRNA - low vs high	K-S test	8.0375E-04
Fig. 4-fig. S7A	trisomy13 cis mRNA - medium vs high	K-S test	1.1507E-07
Fig. 4-fig. S7C	trisomy18 cis mRNA - medium vs high	K-S test	1.8874E-15
Fig. 4-fig. S7E	trisomy21 cis mRNA - medium vs high	K-S test	1.4906E-08
Fig. 4-fig. S7A	trisomy13 cis mRNA - low vs medium	K-S test	7.7870E-03
Fig. 4-fig. S7C	trisomy18 cis mRNA - low vs medium	K-S test	4.2391E-03
Fig. 4-fig. S7E	trisomy21 cis mRNA - low vs medium	K-S test	1.8537E-02
Fig. 4-fig. S7B	trisomy13 cis lncRNA - low vs high	K-S test	5.4989E-02
Fig. 4-fig. S7D	trisomy18 cis lncRNA - low vs high	K-S test	4.2252E-02
Fig. 4-fig. S7F	trisomy21 cis lncRNA - low vs high	K-S test	5.1557E-01
Fig. 4-fig. S7B	trisomy13 cis lncRNA - medium vs high	K-S test	3.0545E-01
Fig. 4-fig. S7D	trisomy18 cis lncRNA - medium vs high	K-S test	4.4189E-02
Fig. 4-fig. S7F	trisomy21 cis lncRNA - medium vs high	K-S test	5.7932E-01
Fig. 4-fig. S7B	trisomy13 cis lncRNA - low vs medium	K-S test	4.0345E-06

Fig. 4-fig. S7D	trisomy18 cis lncRNA - low vs medium	K-S test	4.0950E-02
Fig. 4-fig. S7F	trisomy21 cis lncRNA - low vs medium	K-S test	4.5027E-07
Fig. 4-fig. S7A-B	trisomy13 cis low - mRNA vs lncRNA	K-S test	4.2754E-04
Fig. 4-fig. S7C-D	trisomy18 cis low - mRNA vs lncRNA	K-S test	1.2166E-03
Fig. 4-fig. S7E-F	trisomy21 cis low - mRNA vs lncRNA	K-S test	1.1140E-08
Fig. 4-fig. S7A-B	trisomy13 cis medium - mRNA vs lncRNA	K-S test	7.4920E-02
Fig. 4-fig. S7C-D	trisomy18 cis medium - mRNA vs lncRNA	K-S test	2.1461E-03
Fig. 4-fig. S7E-F	trisomy21 cis medium - mRNA vs lncRNA	K-S test	9.5764E-02
Fig. 4-fig. S7A-B	trisomy13 cis high - mRNA vs lncRNA	K-S test	4.4374E-03
Fig. 4-fig. S7C-D	trisomy18 cis high - mRNA vs lncRNA	K-S test	2.6918E-03
Fig. 4-fig. S7E-F	trisomy21 cis high - mRNA vs lncRNA	K-S test	9.7453E-04
Fig. 4-fig. S8A	trisomy13 trans mRNA - low vs high	K-S test	0.0000E+00
Fig. 4-fig. S8C	trisomy18 trans mRNA - low vs high	K-S test	0.0000E+00
Fig. 4-fig. S8E	trisomy21 trans mRNA - low vs high	K-S test	0.0000E+00
Fig. 4-fig. S8A	trisomy13 trans mRNA - medium vs high	K-S test	0.0000E+00
Fig. 4-fig. S8C	trisomy18 trans mRNA - medium vs high	K-S test	0.0000E+00
Fig. 4-fig. S8E	trisomy21 trans mRNA - medium vs high	K-S test	0.0000E+00
Fig. 4-fig. S8A	trisomy13 trans mRNA - low vs medium	K-S test	0.0000E+00
Fig. 4-fig. S8C	trisomy18 trans mRNA - low vs medium	K-S test	0.0000E+00
Fig. 4-fig. S8E	trisomy21 trans mRNA - low vs medium	K-S test	0.0000E+00
Fig. 4-fig. S8B	trisomy13 trans lncRNA - low vs high	K-S test	3.9857E-14
Fig. 4-fig. S8D	trisomy18 trans lncRNA - low vs high	K-S test	0.0000E+00
Fig. 4-fig. S8F	trisomy21 trans lncRNA - low vs high	K-S test	0.0000E+00
Fig. 4-fig. S8B	trisomy13 trans lncRNA - medium vs high	K-S test	5.9286E-14
Fig. 4-fig. S8D	trisomy18 trans lncRNA - medium vs high	K-S test	0.0000E+00
Fig. 4-fig. S8F	trisomy21 trans lncRNA - medium vs high	K-S test	0.0000E+00
Fig. 4-fig. S8B	trisomy13 trans lncRNA - low vs medium	K-S test	0.0000E+00
Fig. 4-fig. S8D	trisomy18 trans lncRNA - low vs medium	K-S test	0.0000E+00
Fig. 4-fig. S8F	trisomy21 trans lncRNA - low vs medium	K-S test	0.0000E+00
Fig. 4-fig. S8A-B	trisomy13 trans low - mRNA vs lncRNA	K-S test	0.0000E+00
Fig. 4-fig. S8C-D	trisomy18 trans low - mRNA vs lncRNA	K-S test	0.0000E+00
Fig. 4-fig. S8E-F	trisomy21 trans low - mRNA vs lncRNA	K-S test	0.0000E+00
Fig. 4-fig. S8A-B	trisomy13 trans medium - mRNA vs lncRNA	K-S test	0.0000E+00
Fig. 4-fig. S8C-D	trisomy18 trans medium - mRNA vs lncRNA	K-S test	0.0000E+00
Fig. 4-fig. S8E-F	trisomy21 trans medium - mRNA vs lncRNA	K-S test	0.0000E+00
Fig. 4-fig. S8A-B	trisomy13 trans high - mRNA vs lncRNA	K-S test	0.0000E+00
Fig. 4-fig. S8C-D	trisomy18 trans high - mRNA vs lncRNA	K-S test	0.0000E+00
Fig. 4-fig. S8E-F	trisomy21 trans high - mRNA vs lncRNA	K-S test	0.0000E+00
Fig. 5A	chrX all genes - XO/XX vs XY/XX	K-S test	3.5310E-03
Fig. 5A	chrX all genes - XO/XX vs XXY/XX	K-S test	8.9655E-06
Fig. 5A	chrX all genes - XY/XX vs XXY/XX	K-S test	7.8900E-06
Fig. 5A	chrX all genes - XO/XX vs XXY/XY	K-S test	9.1982E-07
Fig. 5A	chrX all genes - XY/XX vs XXY/XY	K-S test	1.7250E-10

Fig. 5B	autosome all genes - XO/XX vs XY/XX	K-S test	0.0000E+00
Fig. 5B	autosome all genes - XO/XX vs XXY/XX	K-S test	0.0000E+00
Fig. 5B	autosome all genes - XY/XX vs XXY/XX	K-S test	0.0000E+00
Fig. 5B	autosome all genes - XO/XX vs XXY/XY	K-S test	0.0000E+00
Fig. 5B	autosome all genes - XY/XX vs XXY/XY	K-S test	0.0000E+00
Fig. 5C	chrX mRNA - XO/XX vs XY/XX	K-S test	4.7309E-02
Fig. 5C	chrX mRNA - XO/XX vs XXY/XX	K-S test	2.4176E-01
Fig. 5C	chrX mRNA - XY/XX vs XXY/XX	K-S test	2.1037E-01
Fig. 5C	chrX mRNA - XO/XX vs XXY/XY	K-S test	3.7891E-01
Fig. 5C	chrX mRNA - XY/XX vs XXY/XY	K-S test	1.6563E-01
Fig. 5D	autosome mRNA - XO/XX vs XY/XX	K-S test	3.3307E-16
Fig. 5D	autosome mRNA - XO/XX vs XXY/XX	K-S test	9.5024E-13
Fig. 5D	autosome mRNA - XY/XX vs XXY/XX	K-S test	1.3433E-02
Fig. 5D	autosome mRNA - XO/XX vs XXY/XY	K-S test	0.0000E+00
Fig. 5D	autosome mRNA - XY/XX vs XXY/XY	K-S test	0.0000E+00
Fig. 5E	chrX lncRNA - XO/XX vs XY/XX	K-S test	2.9243E-01
Fig. 5E	chrX lncRNA - XO/XX vs XXY/XX	K-S test	2.0961E-05
Fig. 5E	chrX lncRNA - XY/XX vs XXY/XX	K-S test	1.5809E-05
Fig. 5E	chrX lncRNA - XO/XX vs XXY/XY	K-S test	1.5674E-08
Fig. 5E	chrX lncRNA - XY/XX vs XXY/XY	K-S test	5.8376E-13
Fig. 5F	autosome lncRNA - XO/XX vs XY/XX	K-S test	4.4020E-12
Fig. 5F	autosome lncRNA - XO/XX vs XXY/XX	K-S test	0.0000E+00
Fig. 5F	autosome lncRNA - XY/XX vs XXY/XX	K-S test	0.0000E+00
Fig. 5F	autosome lncRNA - XO/XX vs XXY/XY	K-S test	0.0000E+00
Fig. 5F	autosome lncRNA - XY/XX vs XXY/XY	K-S test	0.0000E+00
Fig. 5G	chrX all genes - XX/XY vs XXY/XY	K-S test	1.2821E-07
Fig. 5G	chrX all genes - XXXY/XY vs XXY/XY	K-S test	0.0000E+00
Fig. 5G	chrX all genes - XXXXY/XY vs XXY/XY	K-S test	6.6613E-16
Fig. 5G	chrX all genes - XXXY/XY vs XXXXY/XY	K-S test	4.4195E-05
Fig. 5H	autosome all genes - XX/XY vs XXY/XY	K-S test	0.0000E+00
Fig. 5H	autosome all genes - XXXY/XY vs XXY/XY	K-S test	0.0000E+00
Fig. 5H	autosome all genes - XXXXY/XY vs XXY/XY	K-S test	0.0000E+00
Fig. 5H	autosome all genes - XXXY/XY vs XXXXY/XY	K-S test	0.0000E+00
Fig. 5I	chrX mRNA - XX/XY vs XXY/XY	K-S test	1.7458E-04
Fig. 5I	chrX mRNA - XXXY/XY vs XXY/XY	K-S test	1.0285E-10
Fig. 5I	chrX mRNA - XXXXY/XY vs XXY/XY	K-S test	6.1279E-12
Fig. 5I	chrX mRNA - XXXY/XY vs XXXXY/XY	K-S test	3.7880E-01
Fig. 5J	autosome mRNA - XX/XY vs XXY/XY	K-S test	0.0000E+00
Fig. 5J	autosome mRNA - XXXY/XY vs XXY/XY	K-S test	0.0000E+00
Fig. 5J	autosome mRNA - XXXXY/XY vs XXY/XY	K-S test	0.0000E+00
Fig. 5J	autosome mRNA - XXXY/XY vs XXXXY/XY	K-S test	1.4113E-07
Fig. 5K	chrX lncRNA - XX/XY vs XXY/XY	K-S test	2.8385E-03
Fig. 5K	chrX lncRNA - XXXY/XY vs XXY/XY	K-S test	8.5424E-09

Fig. 5K	chrX lncRNA - XXXXY/XY vs XXY/XY	K-S test	1.1366E-05
Fig. 5K	chrX lncRNA - XXXY/XY vs XXXXY/XY	K-S test	3.8536E-01
Fig. 5L	autosome lncRNA - XX/XY vs XXY/XY	K-S test	0.0000E+00
Fig. 5L	autosome lncRNA - XXXY/XY vs XXY/XY	K-S test	0.0000E+00
Fig. 5L	autosome lncRNA - XXXXY/XY vs XXY/XY	K-S test	0.0000E+00
Fig. 5L	autosome lncRNA - XXXY/XY vs XXXXY/XY	K-S test	3.5033E-10
Fig. 6A	trisomy13 - cis-TF vs trans-TF	K-S test	3.0743E-01
Fig. 6C	trisomy18 - cis-TF vs trans-TF	K-S test	2.3959E-02
Fig. 6E	trisomy21 - cis-TF vs trans-TF	K-S test	1.8422E-07
Fig. 6A	trisomy13 cis-TF	normality test	7.2979E-03
Fig. 6A	trisomy13 trans-TF	normality test	0.0000E+00
Fig. 6C	trisomy18 cis-TF	normality test	8.8798E-01
Fig. 6C	trisomy18 trans-TF	normality test	0.0000E+00
Fig. 6E	trisomy21 cis-TF	normality test	7.4278E-04
Fig. 6E	trisomy21 trans-TF	normality test	0.0000E+00
